# Therapeutical and Pharmaceutical Notes

**Published:** 1901-10

**Authors:** W. J. Martin

**Affiliations:** Kankakee, Ill.


### THERAPEUTICAL AND PHARMACEUTICAL NOTES.

UNDER THE DIRECTION OF W. J. MARTIN, M.D.C.,
KANKAKEE, ILL.

ACTINOMYCOSIS IN MAN. Barclay (*Bristol Medico-Chirurgical Journal*, March, 1901) has no doubt that actinomycosis is still regarded by many as a rare and infrequent disease in spite of the records. He thinks that because of the obscure and ill-defined symptomatology of the disease, and its close resemblance in clinical features to several much commoner lesions, there is much danger of failing to recognize the condition.

Once implanted the specific organism, which grows on cereals and grasses, spreads and sets up inflammation. Sometimes an ulcer is formed ; at other times a tumor of a pale yellowish color, globular in shape, riddled with holes of a larger or smaller size, containing pus, and very vascular, although it presents a superficial resemblance to the interior of a tuberculous or gummatous deposit. It infiltrates all the tissues, spreading in the intermuscular planes, and even into the muscles, entering into the cancellous tissues of bone and the solid viscera. The growth may be carried by the vascular channels as emboli, and deposited in distant parts of the body.

The percentage for different parts or regions seems to be as follows : Head and neck, 55 per cent.; digestive tract, 19 per cent.; pulmonary, 14 per cent.; skin, 2 per cent., and doubtful, 5 per cent.

In the jaw it generally simulates the common periosteal or alveolar inflammations, or, occasionally, suppuration in the antrum. One swelling follows another with great inveteracy; sinuses are very obstinate, and induration or infiltration is apt to occur widely in either an upward or downward direction.

Ruhrah lays great stress on the absence of involvement of the lymphatics, which he describes as a constant feature of the disease. There is an early appearance of trismus, which may be attributed to an involvement of the muscles of mastication, although in some cases the temporomaxillary joint is affected. In a case observed by Hoover during life it was impossible to show the presence of streptothrix, but at the autopsy very wide-spread disease was found.

Oliver has described a case where the disease appeared as an ulceration of the mucous membrane of the mouth. They were curetted and healed, leaving a white indurated patch. A purulent affection of the gums appeared coincidently; the teeth were removed with the exception of two, to which artificial ones were clamped. An ulcer appeared opposite the clamp in the right cheek and spread rapidly. No streptothrix or tubercle bacilli were found in the scrapings. Iodide of potassium was given in large doses for two months, but the ulceration only extended further and became very painful. While an operation for swellings that had appeared was being considered, submaxillary swelling softened. On excision a thin, greenish-yellow fluid, containing some yellowish-white curdy masses, was discharged. The microscope failed to show the streptothrix. A radical operation was undertaken, and several weeks later a lump under the ear softened, and, on being opened, the same greenish fluid was evacuated, in which, after a prolonged search, the streptothrix were found. The patient finally died of arterial hemorrhage, probably from the lingual artery.

Cases of this affection involving the skin are quite rare, but Leser describes two forms—ulceration, and a discrete nodular skin inflammation—with central cicatrization and peripheral extension as in lupus. He considers the absence of lymphatic involvement almost pathognomonic, and he says the board-like indurations and the grossly nodular conditions which are found in these cases are not usual in the other affections. The disease may also be mistaken for rodent ulcer when it occurs in the skin.

The only absolutely diagnostic feature is the discovery of sulphur granules in the discharge from sinuses or abscesses, or in the sputum or stools. Great stress has been laid by some observers on the absence of involvement of the neighboring lymphatics in all cases. Godlee says the character of the abscess, as determined by the finger, would make him almost certain of its nature. There are indefinite spongy walls, easily breaking before the finger, and bleeding very freely. Ruhrah says the back of a patient of Black's was a seat of numerous sinuses, whose puffed and reddened openings, together with living intervening skin, gave a perfect picture of actinomycosis, and Volkmann, Bostrom, and Bernhardt believe that the reddening of the skin, turning later to a violent blue tint, shading from the centre to the periphery, is diagnostic of the disease.

The prognosis seems to depend on the possibility of a radical extirpation of the diseased growth, and on the presence or absence of secondary infection.

Whenever possible the diseased area should be completely excised, and Ruhrah recommends that after this, where permissible, the area should be thoroughly cauterized. The administration of iodide of potassium should be pushed at the same time, beginning with five or ten grains three times a day, and increasing it to forty or fifty grains thrice daily. For injection into sinuses, etc., a number of substances have been employed—tincture of iodine, carbolic acid, salicylic acid, nitrate of silver solution, and iodoform. The only hopeful treatment seems to consist in excisions or curetting, repeated as often as necessary, and as early as possible, with the internal administration of iodide of potassium up to the limit of the patient's endurance.—*Therapeutic Gazette.*

